# Eco‐Powered Cleanup: Laccase as a Green Catalyst for Tackling Emerging Contaminants

**DOI:** 10.1002/gch2.202500395

**Published:** 2025-11-08

**Authors:** Michael Dare Asemoloye

**Affiliations:** ^1^ Department of Biological & Environmental Sciences Walter Sisulu University Nelson Mandella Drive Mthatha Eastern Cape 5117 South Africa

**Keywords:** biocatalyst, clean technology, environmental conservation, green remediation, multicopper oxidases, enzyme immobilization

## Abstract

The widespread Presence of emerging contaminants (ECs) from pharmaceuticals, personal care products, and industrial, agricultural, and urban chemicals/wastes has escalated into a pressing global health concern. Key ECs include per‐ and polyfluoroalkyl substances (PFAS), microplastics, certain nanomaterials, endocrine disrupting compounds, and pesticides spanning diverse chemical classes, with harmful implications for humans, animals, and the environment. They have been detected in groundwater, surface water, soils, and wastewaters in different concentrations. Bioremediation has been well praised as a green, ecofriendly method among other methods for environmental remediation. Laccase (Lac), a versatile oxidative enzyme, is distinguished by its ability to act on non‐phenolic substrates, thereby expanding its utility in EC breakdown. This review delves into the origins of ECs and investigates the pivotal role of Lac in their degradation. Lac is one of the most powerful natural oxidative enzymes and is presently receiving the attention of the science community as an effective and versatile green catalyst for eco‐powered cleanup of various contaminants. This review analyses the complex mechanisms behind Lac‐mediated degradation and underscores its promise in promoting sustainable water/land resource management. While its wide use still faces different challenges, innovative methodologies such as Lac immobilization are highlighted as effective approaches for enhancing EC removal and advancing environmental conservation. In essence, the review spotlights the ecological implications of Lac in bioremediation and the transformative approaches for its sustainable applications. Through cutting‐edge techniques and strategic enzyme deployment, this review offers a forward‐looking perspective on Lac in mitigating EC‐induced environmental challenges.

## Graphical abstract

This review explores laccase (Lac) as an eco‐powered green catalyst for degrading persistent emerging contaminants (ECs), threatening ecosystems and human health. Highlighting Lac's oxidative versatility, it examines mechanisms, immobilization strategies, and sustainable applications. Emphasizing innovation and future perspectives, the review positions Lac‐driven bioremediation as a transformative approach for cleaner water systems and global environmental resilience.

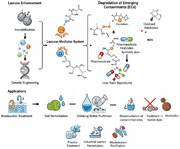

## 1 Introduction

Rising pollution, climate instability, and the depletion of natural resources have amplified global urgency for sustainable practices. Communities are increasingly placing sustainability at the forefront to mitigate the adverse effects of pollution on ecosystems and human health.^[^ ^1^, ^2^ ^]^ The rapid expansion of industrial operations, intensive agriculture, and urbanization has introduced a multitude of pollutants into the environment, many of which are marked by high toxicity, resistance to degradation, and long‐term persistence.^[^ ^3^, ^4^ ^]^ Today, continual detection of emerging contaminants (ECs) in environmental matrices has raised substantial concern due to their potential toxicological impacts on human populations and aquatic ecosystems. ECs encompass a diverse array of compounds, generated from incomplete degradation of wastes from pharmaceuticals (antibiotics, painkillers, and antidepressants), personal care products (sunscreens, fragrances, and preservatives), industrial chemicals (flame retardants, plasticizers, phthalates, solvents), synthetic pesticides and herbicides, plastics, and fibre materials. Although they are present in microgram quantities, their persistence, bioaccumulative nature, and limited regulation contribute to mounting challenges in environmental monitoring and remediation. They are challenging to detect or eliminate, many of which are classified as Group 1 carcinogens by the World Health Organization.^[^ ^5^ ^]^ Their widespread presence in various environmental compartments, coupled with concerns over human exposure and ecological disruption, has become a critical priority in contemporary environmental science, intensifying global interest in effective treatment strategies.^[^ ^3^ ^]^ Conventional physical and chemical methods such as photodegradation, Fenton reactions, electrochemical treatment, ozonation, thermal degradation, and advanced oxidation processes have been employed to mitigate ECs.^[^ ^6^ ^]^ However, these approaches often face significant drawbacks, including high operational costs, energy‐intensive requirements, and the formation of toxic byproducts.

In response to these limitations, the development of innovative, efficient, and eco‐conscious remediation technologies has emerged as a central objective for researchers and environmental professionals. Within this context, ‘Bioremediation’ offers a promising solution.^[^ ^4^ ^]^ As a naturally driven and low‐impact process, it aligns with principles of sustainability and cost‐effectiveness, making it a viable alternative for large‐scale pollutant removal and environmental restoration. Recently, microbial enzymatic degradation has emerged as a promising and environmentally friendly approach for EC treatment.^[^ ^7^ ^]^ Microorganism‐derived enzymes serve as potent biological agents in both the transformation and breakdown of various contaminants. Among these, Laccases (Lacs), members of the multicopper‐containing phenol oxidase family with EC number 1.10.3.2, are widely acclaimed for their potent oxidative capacity and broad substrate specificity, enabling them to catalyse the transformation of a diverse array of organic pollutants.^[^ ^8^, ^9^, ^10^, ^11^ ^]^ In recent years, the application of Lacs in bioremediation and wastewater treatment has gained significant momentum, driven by growing recognition of their ability to break down recalcitrant contaminants and improve the overall efficiency of biodegradation processes.^[^ ^12^, ^13^, ^14^, ^15^ ^]^ Lac mediated oxidation of toxic compounds/chemicals has emerged as a sustainable alternative to conventional chemical remediation strategies, demonstrating efficacy in the removal of industrial pollutants and ECs through enzymatic means. This eco‐friendly approach not only reduces chemical dependency but also aligns with the goals of green chemistry and environmental sustainability.^[^ ^16^ ^]^

This review positions the exploration of Lacs for the degradation of ECs; their powerful innovation, backed by some compelling science. The concept of “Eco‐Powered Cleanup” using Lac centres on leveraging this naturally occurring enzyme to break down ECs pollutants that traditional water treatment methods often fail to remove. Lacs in general use oxygen and produce water as a byproduct with no harmful residues. They have a broad substrate range covering the degradation of both phenolic and non‐phenolic compounds, especially with mediators. Their versatile sources encompass fungi, bacteria, plants, and insects, with the ability to catalyse the oxidation of a wide range of phenolic and non‐phenolic compounds through a four‐electron reduction of oxygen to water. This represents a crucial mechanistic insight that is being harnessed for developing highly efficient and environmentally friendly sustainable remediation applications. A key example is the decolorization of industrial dyes and the degradation of persistent organic pollutants using Lacs. Studies have revealed that Lac can degrade ECs like antibiotics, hormones, and synthetic dyes, reducing their environmental impact. Immobilized Lac systems enhance stability and reusability, making them ideal for scalable water treatment. This aligns with green chemistry principles and supports sustainable water resource management, reducing reliance on energy‐intensive or chemical‐heavy treatment methods.

## 2 Emerging Contaminants and Eco‐Enzyme Innovation for their removal

### 2.1 Emerging Contaminants, their Effects on Humans and the Environment

As societies advance toward highly urbanized infrastructures, anthropogenic activities have intensified, bringing about rapid industrial growth and economic development. This expansion, however, comes at the cost of extensive natural resource exploitation, resulting in significant waste management challenges and the proliferation of environmental pollutants.^[^ ^17^, ^18^ ^]^ The global production of ECs has steadily increased across both developed and developing regions over recent decades, largely due to their persistent and bioactive nature. Various forms of emissions stemming from manufacturing, human activity, and agricultural discharge continue to affect the integrity of air, water, and soil ecosystems (**Table** **1**). Substances such as pesticides, hormones, personal care products, pesticides, pharmaceutical residues, and synthetic textile dyes, microplastics, and microfibers have been strongly associated with neurological, hormonal, and reproductive disorders in humans and animals.^[^ ^19^ ^]^ These compounds are routinely released into aquatic environments through runoff and wash‐off from residential areas, healthcare facilities, and agricultural land, leading to contamination of surface waters and adjoining soils (Table 1).

**Table 1:** Some frequently detected emerging contaminants in water bodies with their molecular formula and molar mass, and their effects on human health.

Contaminant	Molecular Formula	Source	Environmental Persistence	Health Effects	Regulatory Interest
Atrazine	C_8_H_14_ClN_5_	Agricultural runoff from corn and sorghum fields; herbicide application	Highly persistent in soil and water	Endocrine disruption, reproductive toxicity, and a potential carcinogen	EPA‐regulated pesticide; EU banned
Bisphenol A (BPA)	C_15_H_16_O_2_	Leaching from plastic containers, epoxy resins, landfill runoff, and industrial effluents	Medium to high; bioaccumulative	Hormonal imbalance, fertility effects, and increased cancer risk	Listed in many toxic substance inventories
Carbamazepine	C_15_H_12_N_2_O	Human excretion (used for epilepsy and bipolar disorder); wastewater effluent	Highly persistent in aquatic environments	Neurological toxicity, liver stress	Detected in global surface and drinking water
Diclofenac	C_14_H_11_Cl_2_NO_2_	Human and veterinary use; topical gels washed off during bathing; hospital waste	Moderate to high	Kidney and liver toxicity, endocrine disruption	EU Watch List contaminant
Sulfamethoxazole	C_10_H_11_N_3_O_3_S	Antibiotic use in humans and livestock; excretion; wastewater discharge	Persistent; promotes antimicrobial resistance	Antibiotic resistance, allergic reactions	Monitored in wastewater globally
Triclosan	C_12_H_7_Cl_3_O_2_	Antibacterial soaps, toothpaste, cosmetics, washed down household drains	Moderately persistent; lipophilic	Thyroid dysfunction, antimicrobial resistance	FDA & EU regulations for personal care use
Caffeine	C_8_H_10_N_4_O_2_	Beverages, food waste, human excretion, and domestic wastewater	Readily biodegradable, but often detected	Cardiac stimulation, neurological effects	Often used as a marker for domestic wastewater
Sucralose	C_12_H_19_Cl_3_O_8_	Artificial sweeteners in food and drinks; human excretion; wastewater effluent	Very persistent in wastewater	Gut microbiome alteration, uncertain chronic impacts	Growing scrutiny for water quality monitoring
Methylparaben	C_8_H_8_O_3_	Preservatives in cosmetics and pharmaceuticals; washed off the skin into drains.	Low to moderate; often detected	Endocrine disruption, breast cancer links	Restricted in some cosmetic formulations
Estrone	C_18_H_22_O_2_	Natural estrogen from human and animal waste, excretion, and agricultural runoff	Persistent; estrogenic activity	Feminization effects, reduced male fertility, thyroid disruption	Monitored endocrine disruptors in surface water
Perfluorooctanoic acid (PFOA)	C_8_HF_15_O_2_	Industrial use in non‐stick cookware, stain‐resistant fabrics, and firefighting foams	Extremely persistent; bioaccumulative	Liver damage, immune system effects, developmental toxicity, and potential carcinogen	Listed under PFAS regulations; phased out in many countries
Lead	Pb	Old pipes, paints, industrial emissions, and battery waste	Persistent in soil and water	Neurotoxicity, developmental delays in children, and kidney damage	Strictly regulated in drinking water and consumer products
Microplastics	Varies (polymer‐based)	Breakdown of larger plastic waste, synthetic textiles, and personal care products	Persistent; accumulates in ecosystems	Inflammation, oxidative stress, and potential endocrine disruption	Increasing global monitoring and research focus
Polycyclic Aromatic Hydrocarbons (PAHs)	Varies (e.g., C_16_H_10_ for benzo[a]pyrene)	Incomplete combustion of fossil fuels, industrial discharge, and urban runoff	Persistent, lipophilic, and bioaccumulative	Carcinogenic, mutagenic, respiratory, and skin irritation	EPA priority pollutants; monitored in air, water, and soil
Mercury	Hg	Coal combustion, mining, industrial waste, broken thermometers	Persistent; methylmercury bioaccumulates	Neurological damage, developmental toxicity, and immune dysfunction	Global regulation under the Minamata Convention
Phthalates (e.g., DEHP)	C_24_H_38_O_4_	Plasticizers in PVC, cosmetics, and food packaging	Moderate; widespread in the environment	Endocrine disruption, reproductive toxicity, and developmental effects	Regulated in toys, cosmetics, and food contact materials
Chlorpyrifos	C_9_H_11_Cl_3_NO_3_PS	Agricultural pesticide; crop spraying	Persistent in soil and water	Neurodevelopmental effects, respiratory issues	Banned or restricted in several countries
Tetracycline	C_22_H_24_N_2_O_8_	Antibiotic use in livestock and humans: excretion	Moderately persistent; promotes resistance	Antimicrobial resistance, allergic reactions	Monitored in veterinary and wastewater contexts
Nonylphenol	C_15_H_24_O	Industrial detergents, surfactants, and plastic production	Persistent; estrogenic activity	Hormonal disruption, aquatic toxicity	EU restricted under REACH; monitored globally
Hexavalent Chromium (Cr⁶⁺)	Cr⁶⁺	Industrial processes, leather tanning, and electroplating	Persistent in water and soil	Carcinogenic, kidney and liver damage, respiratory issues	Regulated under drinking water standards and industrial discharge

Subsurface infiltration through leaching and filtration processes further allows ECs to reach groundwater systems. In addition to these routes, ECs enter water bodies via disposal of unused medications, wastewater‐based irrigation, landfill leachates, carcass decomposition, and effluent discharge from wastewater treatment plants.^[^ ^5^, ^20^, ^21^, ^22^ ^]^ Solid waste dumping, a common practice in many urban and peri‐urban areas, also exacerbates EC pollution. Recent investigations have revealed the presence of artificial sweeteners (e.g., acesulfame, saccharin, sucralose) in domestic wastewater via human excretion^[^ ^23^ ^]^ and food preservatives such as parabens in groundwater.^[^ ^24^ ^]^ As surface and groundwater remain primary sources of drinking water in many parts of the world, EC contamination raises significant public health concerns. Agricultural runoff has been identified as a key pathway for herbicide residues, particularly atrazine and terbuthylazine, to infiltrate drinking water supplies.^[^ ^25^ ^]^ Additionally, non‐sewered sanitation systems, such as urine‐diverting dry toilets (UDDTs), contribute to EC loads in developed nations. Though limited in coverage, pilot‐scale UDDTs have demonstrated accumulation of several pharmaceuticals, including hydrochlorothiazide, clarithromycin, darunavir, diclofenac, and trimethoprim in source‐separated urine.^[^ ^26^, ^27^ ^]^ Landfill leachate presents another concerning source of EC contamination. Inadequate management and direct discharge of untreated leachates into sewers or surface waters elevate EC concentrations within municipal WWTPs.^[^ ^28^, ^29^ ^]^

These contaminants are commonly found in pharmaceuticals, personal care products, pesticides, and industrial chemicals, and are known for their persistence, bioaccumulation potential, and adverse health effects even at trace concentrations. They typically enter water bodies through wastewater treatment plant effluents, agricultural runoff, leaching from landfills, and stormwater drainage. Many are not fully removed by conventional treatment processes, which is why they persist in surface and groundwater.

ECs pose significant threats to ecosystems and human health due to their persistence and widespread presence in soil and water.^[^ ^17^ ^]^ These pollutants are associated with both acute and chronic effects, including endocrine disruption, immunotoxicity, neurological disorders, and various cancers. ECs are continuously released into the environment through multiple pathways, but inefficient removal and incomplete degradation at treatment plants lead to their long‐term accumulation. Even at extremely low concentrations, ECs can cause serious biological harm, often interacting synergistically in aquatic environments to amplify toxicity. Common ECs such as brominated flame retardants, phthalates, microplastics, and polycyclic siloxanes have been linked to genetic mutations, hormonal imbalance, reproductive disorders, and developmental abnormalities.^[^ ^28^ ^]^ In humans, they are particularly implicated in thyroid dysfunction, feminizing effects in males, and increased risks of cancer and pregnancy complications. These compounds are often resistant to conventional treatment methods and persist in water, soil, and air.^[^ ^29^ ^]^ In response, the need for effective degradation strategies for ECs has grown increasingly urgent.

### 2.2 Green Catalysts for the Degradation of Emerging Contaminants

While traditional chemical and physical treatment methods are available, they often present limitations in cost, efficiency, and ecological impact. This has led to a surge of interest in bioremediation, a promising and environmentally friendly approach that utilizes microorganisms and their enzymatic systems to degrade harmful pollutants. Microbial enzymes, in particular, offer targeted and sustainable pathways to detoxify ECs and restore ecological balance.

Green catalysts are biologically derived enzymes often produced from organic waste or microbial sources that facilitate the breakdown of complex pollutants into less harmful substances. They contain a mix of organic acids, enzymes, and mineral salts, and are typically generated through the fermentation of fruit and vegetable waste.^[^ ^16^ ^]^ Green catalysts, especially those containing oxidoreductases like Lac, peroxidase, and manganese peroxidase, can oxidize and degrade complex organic molecules, reduce toxicity/bioaccumulation potential, and break down endocrine‐disrupting compounds linked to health disorders.^[^ ^29^ ^]^ Enzymatic bioremediation is increasingly recognized as one of the most effective and environmentally sustainable strategies for degrading hazardous compounds, despite the availability of conventional chemical and physical treatment processes.^[^ ^30^, ^31^, ^32^ ^]^ This method leverages the catalytic capabilities of enzyme systems derived from green plants and microorganisms, positioning it as a compelling solution for eliminating ECs from various environmental matrices.^[^ ^16^ ^]^ Recent advances in eco‐enzyme/green catalyst application have focused on improving enzyme performance and stability in three areas: immobilization techniques, which involves anchoring of enzymes with solid supports to enhance reusability and resistance to environmental fluctuations, microbial engineering, tailoring microbes to produce high‐yield, pollutant‐specific enzymes and hybrid systems that combining eco‐enzymes with filtration, adsorption, or advanced oxidation processes for synergistic effects.

Eco‐enzyme innovation aligns with green chemistry principles and supports low‐energy, low‐waste remediation, circular economy models by repurposing organic waste. It also supports community‐level solutions for domestic and industrial wastewater treatment. Among the enzymes employed in this context, oxidoreductases, including lignin peroxidase, manganese peroxidase, Lac, and horseradish peroxidase, are particularly notable for their strong oxidative capacity and effectiveness in decomposing organic pollutants.^[^ ^31^ ^]^ However, despite its promise, enzymatic bioremediation faces several practical challenges, such as enzyme stability, reusability, recyclability, high production costs, and scalability. Of the enzymes applied to wastewater treatment, peroxidase and Lac are most utilized.^[^ ^21^ ^]^ Lac demonstrates superior performance due to its ability to function across a wide range of temperatures and pH levels, making it especially suitable for diverse and dynamic remediation conditions.

## 3 Laccase as a Green Catalyst for the Degradation of Emerging Contaminants

### 3.1 Laccase Enzyme: Source, Structure, and Characteristics

Lac is a highly adaptable multicopper oxidase enzyme renowned for its capacity to oxidize a broad spectrum of substrates, including both phenolic and non‐phenolic compounds.^[^ ^33^ ^]^ This remarkable versatility renders it a valuable tool across diverse environmental and industrial domains. Lacs are naturally produced by a range of biological organisms, plants, fungi, bacteria, and insects. Among these, fungal Lacs have received the most extensive attention due to their wide substrate specificity and robust operational performance.^[^ ^4^ ^]^ Although bacterial, plant, and insect‐derived Lacs are less studied, they exhibit promising attributes such as increased stability under extreme pH and temperature conditions, making them attractive candidates for specialized applications.^[^ ^9^, ^34^ ^]^

Lacs possess unique structural and catalytic attributes that enable participation in a broad spectrum of biochemical transformations. Structurally, lacs are broadly categorized into three types: blue, white, and yellow based on their origin and spectral properties. Among these, blue Lacs are the most common, distinguished by their intense coloration resulting from a Type 1 (T1) copper site that facilitates electron transfer.^[^ ^35^, ^36^ ^]^ Bacterial Lacs are further classified into two structural subgroups: the conventional three‐domain Lacs and the more compact two‐domain variants, each exhibiting distinct architectural and functional traits.^[^ ^35^ ^]^ A hallmark feature of lacs is their incorporation of multiple copper ions arranged in three key coordination centres: T1, Type 2 (T2), and Type 3 (T3). These sites collectively contribute to the enzyme's catalytic efficiency, enabling diverse oxidation reactions. Typically, the active site contains four copper ions, essential for sequential electron transfer and redox cycling. The T1 centre is responsible for the blue coloration and initiates electron transfer from substrates. Electrons then proceed to the T2/T3 trinuclear cluster, which ultimately reduces molecular oxygen to water.^[^ ^36^, ^37^, ^38^ ^]^ This sophisticated electron relay system allows Lac to catalyse a broad range of oxidative reactions, positioning them as valuable agents in biotechnological applications from bioremediation to industrial processing.

The diverse molecular configurations of Lac and its mechanistic pathways have led to the identification of multiple subtypes, each with distinct operational advantages.^[^ ^1^ ^]^ This diversity underpins their widespread application in industries such as environmental remediation, textile processing, and biosensor development.^[^ ^39^ ^]^

### 3.2 Application of Laccases

Laccase belongs to the broader group of multicopper oxidases (MCOs), a family of enzymes that also includes manganese peroxidase (MnPs), lignin peroxidase (LiPs), and versatile peroxidases (VPLs), each contributing to the oxidative breakdown of complex compounds.^[^ ^31^ ^]^ These enzymes are classified across four key families: Lacs (EC 1.10.3.2), ascorbate oxidases (EC 1.10.3.3), ferroxidases (EC 1.16.1), and ceruloplasmin (EC 1.16.1). Lac activity operates through a radical‐based oxidation mechanism, beginning with substrate oxidation at the T1 site, followed by electron shuttling to the T2/T3 cluster, and culminating in oxygen reduction.^[^ ^36^, ^40^ ^]^ Species‐specific structural variations influence substrate specificity and operational stability, further enhancing the versatility of these enzymes across environmental and industrial sectors. A hallmark of Lac functionality lies in its capacity to oxidize a wide range of phenolic and non‐phenolic substrates, including polycyclic aromatic hydrocarbons, synthetic dyes, and pesticides. Such broad substrate specificity not only highlights their catalytic versatility but also reinforces their potential as powerful tools for pollution mitigation and industrial innovation.^[^ ^41^ ^]^

Microbial Lacs are especially notable for their varied physicochemical properties and resilience to inhibitors, which have broadened their adoption in industries including textile processing, wastewater treatment, and environmental remediation.^[^ ^36^, ^37^ ^]^ Fungal Lacs, particularly those from *Aspergillus* species, have demonstrated effective decolorization of industrial textile effluents and notable biocatalytic utility in other sectors. Beyond waste management, Lac's lignolytic activity has enabled its widespread use in the food industry, especially in baking and packaging applications.^[^ ^9^, ^42^, ^43^ ^]^ Its ability to break down contaminants such as phenols and synthetic dyes has positioned Lac as a pivotal agent in ecological conservation and sustainable pollution control.

White rot fungi secrete Lac extracellularly onto plant material, enabling them to break down lignin and support growth. This discovery has since been harnessed by the pulp and paper industry for detoxification and decolorization processes, owing to Lac's ability to oxidize a wide spectrum of phenolic and non‐phenolic compounds. More recently, Lac has gained prominence in environmental biotechnology, particularly in the biodegradation of organic contaminants. Functionally, Lac operates as an electron acceptor and is distinguished by its broad substrate specificity regarding electron donors. It readily catalyzes oxidation reactions involving monophenols and diphenols, and also acts on monolignols, polyphenols, hydroxyindoles, and polyamines, each exhibiting different redox potentials.^[^ ^46^, ^47^ ^]^ This wide reactivity allows Lac to participate in numerous biological and industrial processes. The enzyme initiates oxidation by generating phenoxy radicals, which are highly reactive intermediates. These radicals often polymerize non‐enzymatically, forming complex polymeric structures, a mechanism particularly useful in environmental applications like pollutant immobilization and wastewater treatment.^[^ ^21^, ^44^ ^]^

### 3.3 Laccase: Mechanism of Action

Growing research interest in laccase‐based bioremediation has prompted deeper investigation into the enzyme's interaction mechanisms with organic pollutants. Lacs are copper‐containing oxidoreductases that extract electrons from substrates, facilitating their transfer through protein‐bound functional groups toward a trinuclear copper cluster (TNC). This electron migration culminates with the fourth electron, where molecular oxygen binds and is reduced to water. The reduction of O_2_ to H_2_O is assisted by aspartic and glutamic acids, which donate the necessary hydrogen atoms.^[^ ^45^ ^]^ Structurally, Lac consists of 200–800 amino acids, with a molecular weight ranging between 50–140 kDa, and has been identified in over 7000 microbial species.^[^ ^46^ ^]^ Enzymatic performance varies depending on the source organism, influencing substrate specificity and oxidation efficiency. Notably, the glycosylated carbohydrate regions enhance enzymatic stability and shield the protein from proteolytic degradation.^[^ ^21^ ^]^

Lacs with high redox potential (E_0_) are preferred for treating wastewater pollutants with similarly high E_0_ values, such as phenols and azo dyes.^[^ ^15^ ^]^ Catalytic activity is governed by four copper atoms categorized into three spectroscopically distinct types: i) Type 1 (T1): High E_0_; absorbs at 610 nm, ii) Type 2 (T2): Non‐paramagnetic; lacks strong absorbance, and iii) Type 3 (T3): Absorbs at 330 nm.^[^ ^46^ ^]^ The T1 center, connected to two histidines, one cysteine, and one methionine, initiates electron transfer from the pollutant. Electrons are then relayed to the TNC cluster, composed of T2 and two T3 copper atoms, each ligated by multiple histidines. This configuration facilitates molecular oxygen reduction and determines substrate docking and catalytic specificity. During pollutant remediation, Lac catalyses the formation of unstable phenoxy radicals through electron extraction, particularly from phenolic substrates, which is influenced by ambient pH. These radicals can undergo non‐enzymatic polymerization, contributing to pollutant immobilization. In detail, the mechanism of phenoxy radical polymerization by Lac contributes to pollutant immobilization in remediation by converting soluble, often toxic, phenolic compounds into insoluble, high‐molecular‐weight polymeric products. For example, Lac catalyses the one‐electron oxidation of phenolic pollutants (e.g., simple phenols, chlorophenols, dyes). This process generates highly reactive, transient phenoxy radicals (ArO⋅). These phenoxy radicals spontaneously react and couple with each other and/or with natural organic matter (like humus) already present in the soil or wastewater. This coupling reaction leads to the formation of larger, stable biopolymers that have significantly lower solubility in water. Because the pollutants are now chemically bound and insoluble, their mobility (leaching) is drastically reduced, and their toxicity is typically lessened, effectively immobilizing them within the treatment matrix (e.g., soil or sludge). This makes the contaminated site or wastewater much safer. Electron transfer by Lac reduces Cu^2^⁺ to Cu⁺, which is re‐oxidized as it donates electrons to O_2_, completing the catalytic cycle and forming H_2_O at the TNC cluster. These depictions highlight the connectivity between Cu centres, the role of key amino acids in electron shuttling, and the overall oxidative mechanism underlying laccase‐based bioremediation.

**Figure 1:**
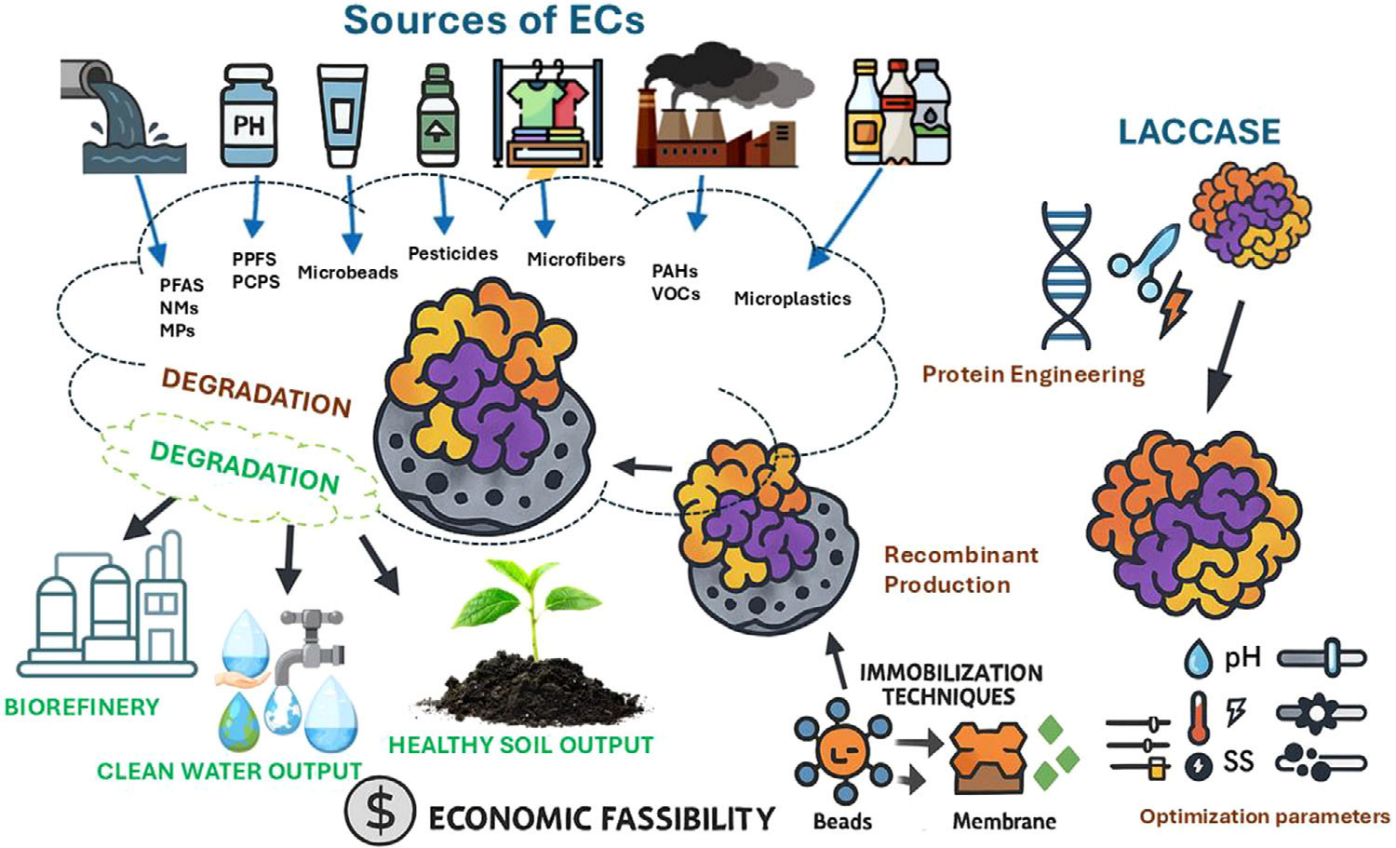
Enhancement and application of laccase for degrading emerging contaminants (ECs), including the enhancement strategies, types of contaminants, degradation pathways, and environmental outcomes. ECs captured here include polycyclic aromatic hydrocarbons (PAHs), volatile organic compounds (VOCs), nanomaterial (NMs), per‐ and polyfluoroalkyl substances (PFAS), Per‐ & poly‐fluoroalkyl substances, pharmaceutical and personal care products (PCPs), and microplastics (MPs).

Despite its promising catalytic capabilities, the application of Lac in pollutant degradation is constrained by several factors. These include limited stability, reduced reusability, and sensitivity to pH and temperature fluctuations. These limitations pose significant hurdles for large‐scale implementation (**Figure** **1**). To address these challenges, enzyme immobilization techniques have emerged as a practical solution. It is enhancing Lac's operational stability, enabling repeated use, and expanding its functional range under diverse environmental conditions.

## 4 Enhancing Operational Stability and Reusability of Laccases through Immobilization Techniques

The efficiency of enzymatic bioremediation is closely tied to the enzyme's operational stability and durability under practical conditions. While free Lacs exhibit impressive capabilities in degrading recalcitrant pollutants, their application in industrial wastewater treatment remains limited due to high sensitivity to environmental stressors and instability under rigorous operating conditions. Furthermore, free Lacs are non‐recoverable, leading to loss in the liquid medium and rendering them unsuitable for reuse. These challenges significantly hinder their scalability and cost‐effectiveness in continuous treatment processes.^[^ ^47^ ^]^ Immobilization of Lac onto solid supports has proven to be a robust strategy for enhancing its long‐term stability and preserving enzymatic efficiency. By anchoring Lac molecules to surfaces, this approach mitigates the risks of denaturation and inactivation during reaction cycles. Common immobilization methods include adsorption, in which the enzyme adheres physically to substrates such as silica or activated carbon, and covalent bonding, where stable chemical linkages are formed with carriers like agarose or glass beads.^[^ ^48^ ^]^ Another key technique is encapsulation, which involves entrapping the enzyme within protective matrices—such as hydrogels or polyacrylamide gels that shield it from extreme environmental conditions and inhibitory compounds. Immobilization not only maintains the enzyme's 3D structure but also restricts its conformational flexibility, thereby reducing the likelihood of unfolding or functional loss.^[^ ^49^ ^]^ This confinement improves the thermal resilience and pH tolerance of Lac, enabling it to retain high catalytic activity over prolonged operational periods.

Additionally, immobilized Lac offers practical advantages in industrial settings. Its easy separation from reaction mixtures facilitates reuse, supports continuous processing, and prevents enzyme leaching, all while maintaining a consistent microenvironment conducive to optimal performance.^[^ ^50^ ^]^ Ultimately, immobilization significantly extends the functional lifespan of Lac and strengthens its utility in scalable biotechnological and environmental applications.^[^ ^51^ ^]^ Immobilization techniques offer a viable solution to address the limitations associated with free Lac enzymes, notably their limited stability and lack of reusability in large‐scale operations. By anchoring Lac to suitable support matrices, enzyme activity and structural integrity are enhanced, allowing for repeated use and sustained performance under industrial conditions, ultimately contributing to cost‐effective bioremediation processes.^[^ ^52^ ^]^

Various carrier materials have been successfully employed to immobilize Lac for the biocatalytic degradation of phenolic and nonphenolic organic pollutants. Notable examples include electrospun poly(methyl methacrylate)/polyaniline fibers,^[^ ^53^ ^]^ Cu‐alginate beads,^[^ ^54^ ^]^ epoxy‐functionalized silica,^[^ ^55^ ^]^ glutaraldehyde cross‐linked chitosan beads,^[^ ^56^ ^]^ and nanofibrous membranes.^[^ ^57^ ^]^ These immobilization platforms have demonstrated improved catalytic efficiency and operational resilience. The ideal properties of carriers for immobilized Lac include chemical, mechanical, and storage stability, biocompatibility, nontoxicity, hydrophilicity, and a large surface area to facilitate enzyme–substrate interaction.^[^ ^58^, ^59^ ^]^ A variety of techniques have been employed for the immobilization of Lacs to enhance their operational performance in bioremediation applications. These include physical adsorption,^[^ ^60^, ^61^ ^]^ ionic adsorption,^[^ ^62^ ^]^ covalent bonding,^[^ ^59^, ^63^, ^64^ ^]^ physical entrapment,^[^ ^51^, ^65^ ^]^ and self‐immobilization through cross‐linked enzyme aggregates (CLEAs).^[^ ^66^, ^67^, ^68^ ^]^ Among these, CLEAs demonstrate high catalytic efficiency due to their dense enzyme clustering, whereas physically entrapped Lacs often suffer from diffusional limitations that restrict their activity to smaller substrates.

Despite this, physical entrapment offers notable reusability and stability, with minimal enzyme leakage. Covalent linking ensures robust enzyme immobilization by forming stable bonds between the enzyme's amino acid residues and functional groups on the carrier matrix, resulting in enhanced stability and resistance to environmental fluctuations.^[^ ^59^, ^69^, ^70^, ^71^ ^]^ To facilitate covalent binding, carrier surfaces are often modified using ionic liquids like[AmimCl] and[Emim][OAc], or hydrophilic cross‐linkers such as N,N′‐methylenebisacrylamide and glycidyl methacrylate.^[^ ^63^, ^64^ ^]^ Ionic adsorption, though cost‐effective, suffers from low operational stability due to the sensitivity of ionic interactions to environmental changes.^[^ ^62^ ^]^ Likewise, physical adsorption involves weak intermolecular forces such as hydrogen bonds and van der Waals interactions, making the enzyme vulnerable to desorption.^[^ ^61^ ^]^ Still, its economic feasibility, simplicity, and catalytic proximity between substrates and enzymes make it the most widely adopted technique.^[^ ^72^ ^]^ Additionally, physical adsorption can mitigate the diffusional barriers encountered in entrapment‐based systems, allowing better substrate enzyme interaction.^[^ ^73^ ^]^

The catalytic performance of Lac in the biodegradation of organic pollutants is profoundly influenced by the pH of the reaction medium. Immobilization techniques effectively shield the reactive groups at the enzyme's active site from harsh pH fluctuations, thereby preserving catalytic function under non‐optimal conditions.^[^ ^52^, ^58^ ^]^ Similarly, cross‐linking and physical entrapment strategies enhance the thermal stability of Lac by increasing enzyme rigidity and minimizing conformational changes in dynamic environments.^[^ ^7^, ^58^ ^]^ Gel matrices used in entrapment not only stabilize the enzyme structurally but also act as thermal buffers by absorbing ambient heat.^[^ ^74^ ^]^ Immobilization of *Trametes versicolor* Lac onto Fe_3_O_4_@SiO_2_‐chitosan nanoparticles has demonstrated high relative activity and resilience across a broad spectrum of pH and temperature ranges.^[^ ^59^ ^]^ Similarly, improved catalytic efficiency was observed in the degradation of bisphenol A, attributed to the protective effects of alginate gel cross‐linking, which safeguards key catalytic amino acids.^[^ ^57^ ^]^

Despite these advantages, immobilized Lacs face several limitations. These include low enzyme loading efficiency, restricted mass transfer, limited substrate accessibility, and potential enzyme leaching, all of which can reduce overall activity.^[^ ^65^ ^]^ Excessive enzyme loading may lead to agglomeration on carrier surfaces, obstructing substrate access to active sites and diminishing catalytic output.^[^ ^59^, ^75^ ^]^ Moreover, the concentration of glutaraldehyde, a common cross‐linking agent, can significantly affect enzyme performance; high concentrations may induce structural alterations, impeding enzymatic stability.^[^ ^59^ ^]^ The operational longevity of immobilized Lacs is governed by factors such as storage duration, pH and temperature of storage, and the number of reuse cycles. Although a gradual decline in activity occurs with extended storage under varying conditions, immobilized forms generally outperform free enzymes in maintaining functionality. For example, Fe_3_O_4_@SiO_2_‐chitosan‐bound Lac retained 60% relative activity after 14 days at pH ≤ 4.0 and 25 °C.^[^ ^59^ ^]^ Remarkably, the same system sustained over 80% activity across 10 reuse cycles, offering clear economic and operational benefits for large‐scale bioremediation.

## 5 Approaches for Integrating Laccase into Wastewater Treatment Technologies

Lacs are widely recognized for their efficiency in the oxidative degradation and removal of both phenolic and non‐phenolic pollutants from wastewater streams. Recent studies have demonstrated the versatility of immobilized Lacs in tackling persistent organic contaminants. For instance, Lassouane et al.^[^ ^52^ ^]^ employed immobilized lac to degrade bisphenol A, a well‐known endocrine disruptor linked to reproductive dysfunction and certain cancers. Similarly, Wang et al.^[^ ^72^ ^]^ reported the successful immobilization of Lac on alkali‐modified biochar for the degradation of malachite green, a toxic compound associated with mutagenic, carcinogenic, and teratogenic effects.^[^ ^76^ ^]^ The enzyme facilitated malachite green breakdown via demethylation and hydroxylation reactions. To enhance removal efficiency in large‐scale operations, continuous‐mode laccase‐based processes, such as fluidized bed bioreactors, have emerged as suitable systems successfully applied to bisphenol A degradation.^[^ ^52^ ^]^ However, non‐magnetic carriers used in enzyme immobilization often present challenges in such systems, including enzyme loss due to the difficulty of recovering carriers from soil and aqueous environments.^[^ ^59^ ^]^

To overcome these limitations, researchers have turned to magnetic nanocarriers, particularly Fe_3_O_4_ nanoparticles, for enzyme immobilization. These materials enable magnetic separation and reuse, significantly reducing enzyme wastage and improving operational sustainability.^[^ ^51^, ^59^ ^]^ Magnetic Fe_3_O_4_ nanoparticles exhibit desirable structural features. For instance, its adjustable pore sizes, high surface area, and ordered porosity collectively enhance the catalytic performance of Lacs in degrading organic pollutants. A notable development by Deng et al.^[^ ^59^ ^]^ involved the creation of a Fe_3_O_4_@SiO_2_‐chitosan immobilized Lac system, where reactive amino groups on the enzyme were linked to the magnetic core via chitosan, and cross‐linked using glutaraldehyde. The SiO_2_‐coated magnetic particles offer multiple advantages, including biocompatibility, hydrophilicity, and chemical modifiability, as well as tunable shell thickness to optimize enzyme–substrate interactions.

Robust biocatalyst formulations are essential for improving the operational stability and catalytic performance of Lac in degrading highly potent, recalcitrant, and toxic organic pollutants. One promising strategy involves the co‐immobilization of Lac with small‐molecule redox mediators, which enhances the enzyme's redox potential and broadens its substrate scope, including the breakdown of high‐redox aromatic compounds.^[^ ^77^ ^]^ For example, Jia et al.^[^ ^63^, ^64^ ^]^ developed a novel biocatalyst by co‐immobilizing Lac and acetosyringone (AS) on regenerated bacterial cellulose (BC) modified with glycidyl methacrylate (GMA) and dopamine (DA). This formulation, termed Lac‐AS@BC/GMA‐DA, demonstrated superior degradation efficiency for 2,4,5‐trichlorophenol (2,4,5‐TCP), achieving 98.3% removal within 16 h at 30 °C for a 20 mg L^−1^ concentration. This far surpassed the performance of ABTS co‐immobilized Lac in cellulose beads (61.6%) and free Lac (38.2%) under similar conditions. Additionally, Lac‐AS@BC/GMA‐DA effectively reduced the phytotoxicity of 2,4,5‐TCP and proved adaptable in various industrial wastewater contexts, including lake water, coal‐washing effluent, and coking wastewater. Gu et al. (2020) reported that Lac co‐immobilized with ABTS in composite beads exhibited 99.9% and 97.4% degradation for indole and carbazole, respectively, and retained high catalytic efficiency across ten reuse cycles, highlighting the durability of mediator‐assisted Lac systems.

Expanding this concept further, Zhou et al.^[^ ^78^ ^]^ engineered a multifunctional biocomposite by co‐immobilizing Lac within a PEG‐modified covalent triazine framework (CTF) supported by Cu‐doped calcium alginate gel beads, designated as Ca/Cu‐PEG@CTF‐OMe‐lac. This platform offered synergistic photocatalytic and enzymatic degradation of pollutants such as phenol, chlorinated phenols, nonylphenol, bisphenol A, and indole. The photocatalytic activity of CTF under visible light, combined with PEG‐enhanced aqueous dispersion and Cu‐mediated oxidation, provided enhanced stability and pH tolerance. In ABTS radical assays, the system achieved ≥87% removal efficiency for multiple high‐redox phenolic compounds, outperforming Lac alone by a significant margin.

In another innovative approach, Gao et al.^[^ ^79^ ^]^ developed a green and efficient method using vault‐encapsulated Lacs for the detoxification of synthetic dyes such as Reactive Blue 19 and Acid Orange 7. This encapsulation strategy markedly reduced cytotoxicity to bacterial species (*E. coli*, *S. epidermidis*), mitigated chlorophyll inhibition in *Chlorella vulgaris*, and preserved mitochondrial activity in Sf9 insect cells, underscoring the enzyme's biocompatibility and detoxifying potential.

## 6 Engineered Laccase for the Biodegradation of Persistent Micropollutants

### 6.1 Recombinant Laccases as a Landscape for Bioremediation of Emerging Contaminants

Lacs are widely utilized for the oxidative transformation of various environmental pollutants. These include phenolic compounds such as methoxyphenols and polycyclic aromatic hydrocarbons (PAHs), as well as non‐phenolic compounds like aromatic amines, anilines, thiols, pesticides, and synthetic dyes.^[^ ^80^, ^81^, ^82^, ^83^ ^]^ Naturally, Lacs are abundantly produced by fungi, plants, and bacteria, with most characterized enzymes originating from fungal and plant species due to their high yield and catalytic efficiency.^[^ ^84^ ^]^ However, bacterial Lacs are increasingly viewed as promising alternatives for industrial applications, owing to their broader pH tolerance, thermal stability, and enhanced resistance to organic solvents, high salt concentrations, and common Lac inhibitors, surpassing many limitations of fungal enzymes.^[^ ^85^, ^86^ ^]^ For instance, basidiomycete white rot fungi typically produce high‐redox potential Lacs active at low pH, whereas bacterial and ascomycete‐derived Lacs exhibit optimal activity in neutral to alkaline conditions (pH 6.0–9.0), offering flexibility across diverse environmental settings.

While native hosts can yield substantial Lac quantities through production optimization, their intrinsic properties remain fixed. In contrast, recombinant Lac (rLac), generated via heterologous gene expression, allows for custom tailoring of enzyme characteristics, such as improved stability, modified substrate specificity, and enhanced performance under harsh conditions (**Table** **2**). These engineered enzymes can be strategically designed for specific applications, with a particular emphasis on the degradation of persistent micropollutants.^[^ ^87^ ^]^

**Table 2:** Recombinant laccases engineered for bioremediation of emerging contaminants.

Recombinant laccase	host organism	Target contaminants	Engineering strategy	Bioremediation efficiency	Remarks	Refs.
Mixed microbial consortia	*Various engineered strains*	Pharmaceuticals, personal care products, and industrial byproducts	Synthetic biology and metabolic pathway integration	Variable (compound‐specific)	Used in integrated bioremediation systems combining multiple microbial functions	[95]
rLac from *Bacillus sp*.	*Bacillus subtilis*	Xenobiotics (e.g., PAHs, pesticides, dyes)	Genetic engineering for high‐temperature activity and pH tolerance	High (>85%) under optimized conditions	Converts xenobiotics into non‐toxic forms via oxidation and ring‐opening	[96]
rLac from *Bacillus subtilis*	*Bacillus subtilis*	Antibiotics, synthetic hormones	Promoter engineering, secretion pathway tuning	Moderate to high (60–80%)	GRAS status of the host makes it suitable for environmental applications	[97]
rLac‐HhC‐125 from *Halalkalibacterium halodurans*	*Halalkalibacterium halodurans*	Industrial dyes, phenolic compounds	Protein yield optimization, extremophile adaptation	High activity under alkaline and saline conditions	Suitable for harsh industrial wastewater environments	[98]
rLac from *Pseudomonas stutzeri*	*Pseudomonas stutzeri*	Phenolic compounds, dyes, lignin derivatives	Substrate optimization, crop residue‐based induction	High (>80%) in optimized conditions	Cost‐effective production using agricultural waste	[99]
Marine‐derived laccase	*Marine fungi*	Toxic pollutants, endocrine disruptors	Isolation from extreme environments	Moderate to high (varies by compound)	Naturally adapted to saline and variable pH conditions	[97]
Marine‐derived laccase	*Marine fungi*	Endocrine disruptors, toxic pollutants	Isolation from extreme environments	Moderate to high (compound‐dependent)	Naturally adapted to saline and variable pH conditions	[95]
rLac from* Phanerochaete flavido‐alba *	*Aspergillus niger*	PAHs, synthetic dyes, pharmaceuticals	Promoter engineering, secretion optimization	Enhanced lac expression	High (>85%)	[100]
rLac from *Myceliophthora thermophila*	*Pichia pastoris*	Endocrine disruptors, pesticides	Thermostability enhancement	Thermostable lac expressed	High (>90%)	[101]
rLac from *Trichoderma reesei*	*Saccharomyces cerevisiae*	Phenolic compounds, textile dyes	Codon optimization, glycosylation control	High expression in yeast	Moderate to high (60–80%)	[102]
rLac from *Penicillium chrysogenum*	*Escherichia coli*	Pharmaceuticals, industrial pollutants	Directed evolution	Enhance bioremediation	Moderate (50–70%)	[103]
rLac from *Trametes versicolor*	*Pichia pastoris*	Bisphenol A, triclosan, and pharmaceuticals	Codon optimization, signal peptide engineering	High removal (>80%) in lab‐scale reactors	Enhanced secretion and stability in wastewater conditions	[104]
rLac from *Pleurotus ostreatus*	*Escherichia coli*	Dyes, phenolic compounds	Directed evolution, fusion tags	Moderate (50–70%)	Requires co‐expression of chaperones for proper folding	[97]
rLac from *Myceliophthora thermophila*	*Aspergillus niger*	PAHs, endocrine disruptors	Thermostability enhancement	High (>85%) at elevated temperatures	Suitable for industrial effluent treatment	[97]
rLac from *Coprinopsis cinerea*	*Saccharomyces cerevisiae*	Pharmaceuticals, pesticides	Site‐directed mutagenesis	High (70–90%)	Improved substrate specificity and pH tolerance	[105]
rLac from *Hymenoptera spp*. (e.g., bees, wasps)	*Escherichia coli*	Phenolic pollutants, synthetic dyes	Codon optimization, expression tuning	Moderate to high (50–80%)	Naturally adapted to oxidative stress; promising for harsh environments	^–^
Immobilized fungal laccase	*White‐rot fungi*	Herbicides, pharmaceuticals, dyes	Nanoparticle immobilization	Enhanced stability and reusability	Used in microbial fuel cells for dye decolorization	[39]
rLac2 from *Cerrena unicolor* (Mossy maze polypore mushroom)	*Pichia pastoris GS115*	Azoxystrobin, Phoxim (pesticides)	Codon optimization, structural motif enhancement	Very high (96.2% for azoxystrobin)	Exceptional pH and thermal stability; novel catalytic motifs	[106, 107]
Immobilized laccase from Trametes versicolor	*Immobilized on nanoparticles*	Organic dyes, pharmaceuticals	Enzyme immobilization on nanocarriers	High (>85%) with reusability over multiple cycles	Used in wastewater treatment and biosensors	[108]
Immobilized fungal laccase	*Trametes versicolor*	Organic dyes, pharmaceuticals	Cross‐linked enzyme aggregates (CLEAs)	High (>90%) with enhanced reusability	Effective in municipal wastewater treatment systems	[97]
rLac from *Trametes versicolor*	*Pichia pastoris*	Bisphenol A, triclosan, and pharmaceuticals	Codon optimization, secretion signal tuning	High (>80%)	Widely used yeast host for industrial enzyme production	[89]
rLac from *Pleurotus ostreatus*	*Saccharomyces cerevisiae*	Phenolic compounds, dyes	Directed evolution, fusion tags	Moderate (50–70%)	Requires co‐expression of folding chaperones	[109]
rLac from *Cerrena unicolor*	*Pichia pastoris GS115*	Azoxystrobin, Phoxim (pesticides)	Structural motif enhancement	Very high (96.2% for azoxystrobin)	Exceptional pH and thermal stability	[97]
rLac from *Myceliophthora thermophila*	*Yarrowia lipolytica*	PAHs, endocrine disruptors	Thermostability and secretion optimization	High (>85%)	Promising non‐conventional yeast host for high‐yield expression	[109]
rLac from *Trametes hirsuta*	*Pichia pastoris*	Industrial dyes, phenolic pollutants	Codon optimization, secretion signal tuning	High (>80%)	Effective in textile wastewater treatment	[89, 110]
rLac from *Panus tigrinus*	*Saccharomyces cerevisiae*	Pharmaceuticals, synthetic hormones	Directed evolution, glycosylation site modification	Moderate to high (60–85%)	Improved thermostability and substrate specificity	[95]
rLac from *Coriolopsis gallica*	*Yarrowia lipolytica*	Endocrine disruptors, pesticides	Promoter engineering, secretion pathway tuning	High (>90%)	Promising host for high‐yield and stable enzyme production	[111]
rLac from Streptomyces coelicolor	*Pichia pastoris*	Brilliant Blue G and Trypan Blue dye	Gene cloning	High (> 90%) in 6 h	Colour removal	[112]
rLac from *Trametes pubescens*	*Pichia pastoris*	Phenolic compounds, dyes	Codon optimization, secretion signal tuning	High (>80%)	Commonly used in dye decolorization and wastewater treatment	[113]
rLac from *Ganoderma lucidum*	*Saccharomyces cerevisiae*	Pharmaceuticals, endocrine disruptors	Glycosylation site modification	Moderate to high (60–85%)	Known for high redox potential and broad substrate range	[114]
rLac from *Lentinula edodes*	*Pichia pastoris*	Antibiotics, synthetic hormones	Directed evolution	High (>90%)	Enhanced thermostability and pH tolerance	[115]
rLac from *Pycnoporus cinnabarinus*	*Aspergillus niger*	PAHs, pesticides	Promoter engineering, secretion pathway tuning	High (>85%)	Produces laccase with high redox potential suitable for complex pollutants	[104, 114, 116]
rLac from Escherichia coli K12	*Pichia pastoris GS115*	Synthetic dyes	Gene expression	High 96.7% of aniline blue removed	Improved degradation of synthetic dyes	[117]
rLac from *Coleoptera spp*. (e.g., beetles)	*Pichia pastoris*	Agrochemicals, pharmaceuticals	Structural motif enhancement	High (>85%)	Insect laccases show unique substrate specificity and thermal stability	[118]
rLac from *Bombyx mori* (silkworm)	*Escherichia coli*	Phenolic compounds, textile dyes	Codon optimization, expression vector tuning	Moderate to high (60–80%)	Silkworm laccases show high activity under neutral pH	[119]
rLac from Tenebrio molitor (mealworm)	*Pichia pastoris*	Agrochemicals, pharmaceuticals	Signal peptide engineering	High (>85%)	Adapted to oxidative stress; potential for soil remediation	[120]
rLac from *Apis mellifera* (honeybee)	*Saccharomyces cerevisiae*	Endocrine disruptors, synthetic hormones	Directed evolution	Moderate (50–70%)	Naturally occurring laccase‐like oxidases with detoxification potential	[87]
rLac from *Arabidopsis thaliana*	*Escherichia coli*	Phenolic compounds, synthetic dyes	Codon optimization, expression vector tuning	Moderate to high (60–80%)	Bioremediation of hydrocarbon pollution	[121]
rLac from *Populus trichocarpa*	*Pichia pastoris*	Agrochemicals, pharmaceuticals	Promoter engineering, glycosylation control	High (>85%)	Biodegradation of agrochemical wastes	[122]
rLac from *Zea mays* (maize)	*Saccharomyces cerevisiae*	Endocrine disruptors, industrial pollutants	Directed evolution	Moderate (50–70%)	Bioremediation of xenobiotics	[123]
rLac from *Medicago truncatula*	*Escherichia coli*	Phenolic compounds, pharmaceuticals	Codon optimization, expression vector tuning	Moderate to high (60–80%)	Removal of hydrocarbon contaminants	[124]
rLac from *Oryza sativa* (rice)	*Pichia pastoris*	Agrochemicals, endocrine disruptors	Promoter engineering, glycosylation control	High (>85%)	rLacs show unique removal of agrochemical conytaminants	[125]
rLac from *Nicotiana tabacum* (tobacco)	*Saccharomyces cerevisiae*	Industrial pollutants, synthetic dyes	Directed evolution	Moderate (50–70%)	Wastewater treatments	[126]

### 6.2 Yeast System for Biosynthesis of Laccases

As lower eukaryotes, yeast cells offer numerous advantages for the industrial production of recombinant biomolecules, particularly those of eukaryotic origin.^[^ ^88^ ^]^ Their widespread use in Lac production is attributed to several key benefits, including ease of cultivation, low‐cost growth media, rapid cell proliferation, and high genetic tractability. Crucially, yeast systems also support eukaryotic post‐translational modifications, such as proteolytic processing, disulfide bridge formation, and glycosylation, which are essential for proper folding and functional expression of Lac enzymes.^[^ ^89^ ^]^ Moreover, yeast platforms are compatible with advanced protein engineering techniques, including site‐directed mutagenesis, directed evolution, and DNA shuffling. These enable substantial improvements in enzyme yield, thermostability, and tolerance to organic solvents.^[^ ^88^ ^]^ Thus, yeasts are highly attractive hosts for recombinant Lac production aimed at diverse biotechnological and environmental applications. Despite the promising characteristics of recombinant Lacs (rLacs), the development of robust variants suited for industrial‐scale bioremediation remains challenging. Critical bottlenecks such as low expression efficiency, limited substrate specificity, and susceptibility to environmental inhibitors must be addressed to fully realize their potential.

### 6.3 Non‐Yeast System for Biosynthesis of Laccases

For more than 25 years, researchers have been working on recombinant rLac production in non‐yeast systems. Currently, many Lacs have been produced in filamentous fungi, bacteria, and even in insects and plants.^[^ ^90^ ^]^ Bacteria have emerged as effective platforms for Lac production due to their ease of cultivation, low cost, and straightforward handling procedures. This makes them particularly valuable for producing Lacs that are otherwise difficult to extract from native hosts. Notably, *Escherichia coli* has become a widely used system for the heterologous expression of Lacs sourced from both bacterial and fungal organisms.^[^ ^91^ ^]^ However, expression in *E. coli* often results in the formation of recombinant enzyme aggregates, leading to reduced overall yield. To overcome these limitations, several strategies, including site‐directed mutagenesis, random mutational approaches, and oxygen‐limiting cultivation conditions, have been implemented to improve Lac yield and functionality. These engineered bacterial Lacs have shown promising applications in the oxidation of synthetic dyes and the bioremediation of phenolic pollutants, highlighting their potential for industrial and environmental use.^[^ ^92^, ^93^ ^]^

Filamentous fungi present a highly promising platform for the recombinant production of diverse proteins, primarily due to their natural ability to secrete proteins extracellularly. Historically, their application has been limited by the lack of efficient genetic modification tools, restricting exploration to only a few species. However, advancements in molecular biology and transformation technologies have significantly expanded the scope for genetic engineering in these organisms. Notably, Lac from *Trametes versicolor* has been successfully expressed in other fungal hosts such as *Trametes ressei*, *Aspergillus oryzae*, and *Aspergillus niger*, leveraging the secretion pathways and adaptability of these filamentous fungi. Recombinant Lacs produced in these systems have been investigated for critical environmental applications (Table 2), including dye decolorization, phytoremediation of polychlorinated biphenyls (PCBs), and elimination of Aflatoxin B_1_ (AFB_1_), demonstrating their potential as sustainable biocatalysts.^[^ ^82^, ^94^ ^]^

### 6.4 Challenges of Laccase Engineering

The presence of unintended enzymatic side reactions during biocatalysis can pose substantial risks to both reaction outcomes and environmental safety. Enzymes secreted by host organisms that are not tailored to the desired application may interfere with target enzymatic processes or generate harmful byproducts.^[^ ^127^ ^]^ Examples are the natural production of glucoamylase and α‐amylase, as well as various cellulases, endonucleases, and catalases by *Aspergillus niger* and *A. oryzae*.^[^ ^128^ ^]^ A critical limitation of many naturally secreted enzymes is their inability to withstand harsh industrial conditions, such as extreme pH, high salinity, and elevated temperatures. Extensive studies have been conducted to evaluate the tolerance of biocatalysts in such environments, particularly in dye decolorization applications.^[^ ^91^ ^]^ The optimal pH for Lac activity varies based on the specific dye and its concentration, with fungal Lacs typically performing best in the acidic pH range of 3–5. Deviations from this range significantly reduce dye decolorization efficiency.^[^ ^128^, ^129^, ^130^ ^]^ In contrast, bacterial azo‐reductases tend to exhibit optimal activity within pH 6–8 and temperature ranges of 25–45 °C. In another instance, *Bacillus cereus* displays maximal reductase activity at 40 °C and pH 7, whereas *Pseudomonas aeruginosa* operates best at 35 °C and pH 7.^[^ ^131^ ^]^ Such variability poses challenges in designing robust biocatalytic systems that perform consistently across changing environmental conditions. Additionally, prolonged biocatalytic reactions may lead to product repolymerization, particularly in the case of azo dyes, which limits degradation efficiency by maintaining azo group integrity. A further concern in biocatalyst application is the safety profile of the host species. Commercial enzyme preparations must be scrutinized for the presence of mycotoxins and other undesirable metabolites. Host organisms should not release compounds of toxicological concern during catalysis, as this poses a major regulatory and environmental hurdle.^[^ ^132^ ^]^

## 7 Challenges of Laccase Application in Bioremediation and Future Perspective

Recent progress in modern biotechnology, protein engineering, biochemistry, and bioinformatics has significantly accelerated the discovery and development of novel enzymes for biocatalytic applications. Enzymes engineered through computational design demonstrate enhanced performance and can be integrated into multistep reaction cascades, thereby broadening their applicability. Technologies such as genome editing, rapid DNA assembly, pathway refactoring, and high‐throughput screening have enabled the automated production of desirable enzymatic traits in microbial hosts. Nevertheless, recombinant DNA technology remains associated with high costs, long development timelines, and unpredictable results, underscoring the need for more efficient and accessible platforms to reduce the time and financial burden of biocatalyst development.^[^ ^131^ ^]^

Biocatalysts, derived from nature's catalytic repertoire, facilitate chemical transformations through various enzyme‐mediated mechanisms. These systems have proven effective in degrading complex organic pollutants, converting them into environmentally benign intermediates. Among these, enzymes from the oxidoreductase family have shown promise in tackling micropollutants. The use of enzyme‐based strategies offers several advantages, including low sludge production, adaptability to varying pollutant concentrations, energy efficiency, and alignment with sustainability goals. Multiple studies support the efficacy of biocatalytic approaches in pollutant removal and demonstrate their viability in green remediation frameworks.^[^ ^133^, ^134^ ^]^

Within this context, Lac has emerged as a key biocatalyst, with applications spanning industries such as textiles, food, paper, and environmental biotechnology. Its growing market reflects its versatility, and its share within the multi‐million‐dollar biocatalyst sector continues to expand. Despite this commercial momentum, most Lac‐related studies remain confined to laboratory‐scale, controlled environments. To transition toward large‐scale implementation, rigorous economic assessments and real‐world evaluations are essential. Notably, performance in simulated or industrial settings, such as treating textile dye effluents, can diverge significantly from controlled outcomes, posing a major challenge for scalability and feasibility.^[^ ^135^, ^136^ ^]^

Addressing such limitations demands innovative engineering solutions, especially in enhancing Lac's stability, viability, and reusability. Immobilization of Lac has shown promise in resolving some of these challenges, while enzyme engineering techniques continue to offer opportunities to develop more robust variants with extended operational lifespans and higher catalytic efficiencies.

Despite numerous immobilization methods that have been explored and documented in recent literature, establishing a universal protocol remains elusive due to the diverse isoforms of Lac, each characterized by distinct optimal conditions and catalytic behaviours. Furthermore, immobilization introduces its own constraints, such as catalytic activity loss, enzyme leaching, substrate‐enzyme mass transfer limitations, quantification challenges, and the need for costly support materials.^[^ ^137^ ^]^ These barriers must be carefully addressed to optimize the environmental and economic impact of Lac‐based bioremediation strategies. Despite these complexities, both free and immobilized Lacs continue to attract interest due to their pollutant degradation capabilities. The rising volume of scientific publications reflects this momentum. Still, extensive research is required to validate large‐scale applications, assess real‐world performance, and define sustainable pathways for Lac deployment in pollutant mitigation and environmental restoration.

## 8 Conclusion and Way Forward

In the face of escalating contamination from persistent organic pollutants, Lac stands out as an excellent biocatalyst candidate. Lac poses a vital biocatalyst role for breaking down ECs, converting them into less harmful derivatives. Reports of successful EC degradation underscore their potential, though scaling up to pilot‐level bioprocesses depends heavily on economic viability. In addition, Lac‐based biodegradation has gained substantial attention due to the enzyme's broad substrate specificity and versatility in targeting micropollutants. However, associated challenges such as reduced reaction rates and mass transfer limitations must be addressed to optimize performance.

For industrial‐scale applications such as large‐scale wastewater treatment, strategies like enzyme immobilization and protein engineering are particularly promising. Generally, immobilization facilitates enzyme reusability, thereby enhancing economic feasibility. Though synthetic biology and recombinant technologies remain resource‐intensive and unpredictable, the integration of computer‐aided enzyme engineering, coupled with multi‐enzyme cascade design, has begun to streamline the development pipeline and expand functional capabilities. Also, optimizing environmental and operational parameters, such as pH, temperature, suspended solids, and mechanical stress, is essential to maximize enzyme yield and activity. Enhanced adaptability of Lac across diverse environmental conditions, potential for genetic and structural enhancement, and compatibility with sustainable treatment systems position it at the forefront of future green remediation technologies. The current market demands technological innovations that accelerate enzyme development while minimizing costs and complexity.

Future directions for Lac application as a green catalyst for bioremediation of ECs thus rely on enhancement strategies like adequate application of activators, mediators, and immobilization materials. Advances in protein engineering have made their application increasingly feasible. Despite challenges posed by complex wastewater compositions, including high salinity and elevated pH, engineered Lacs offer promising solutions. Machine learning and enzyme engineering to optimize degradation pathways, integration into smart bioreactors for real‐time pollutant monitoring, and policy and public awareness to promote adoption in agriculture, industry, and urban planning. Immobilization of Lacs presents a practical approach for treatment systems, yet the extraction and purification processes remain time‐consuming. Producing recombinant Lacs with enhanced stability and activity could significantly streamline purification, improving cost‐effectiveness and enabling broader commercial application in wastewater treatment.

## Conflict of Interest

The authors declare no conflict of interest.

## Author Contributions

M.D.A. performed conceptualization, formal analysis, investigation, methodology, project administration, validation, visualization, instrumental software, supervision, project administration, resources, validation, formal analysis, acquired funding acquisition, wrote, reviewed, and edited the final manuscript.
